# The Effect of Airway Pressure Release Ventilation on Pulmonary Catheter Readings: Specifically Pulmonary Capillary Wedge Pressure in a Swine Model

**DOI:** 10.1155/2011/371594

**Published:** 2011-02-24

**Authors:** Ahmad M. Slim, Shaun Martinho, Jennifer Slim, Eddie Davenport, Luadino M. Castillo-Rojas, Eric A. Shry

**Affiliations:** Cardiology Service, Brooke Army Medical Center, 3851 Roger Brooke Drive, Fort Sam Houston, TX 78234-6200, USA

## Abstract

*Background*. Airway pressure release ventilation (APRV) is a mode of mechanical ventilation that theoretically believed to improve cardiac output by lowering right atrial pressure. However, hemodynamic parameters have never been formally assessed. *Methods*. Seven healthy swine were intubated and sedated. A baseline assessment of conventional ventilation (assist control) and positive end-expiratory pressure (PEEP) of 5 cm H_2_O was initiated. Ventilator mode was changed to APRV with incremental elevations of CPAP-high from 10 to 35 cm H_2_O. After a 3-to-5-minute stabilization period, measurements of hemodynamic parameters (PCWP, LAP, and CVP) were recorded at each level of APRV pressure settings. *Results*. Increasing CPAP caused increased PCWP and LAP measurements above their baseline values. Mean PCWP and LAP were linearly related (LAP = 0.66^*∗*^PCWP + 4.5 cm H_2_O, *R* ^2^ = 0.674, and *P* < .001) over a wide range of high and low CPAP values during APRV. With return to conventional ventilation, PCWP and LAP returned to their baseline values. *Conclusion*. PCWP is an accurate measurement of LAP during APRV over variable levels of CPAP. However, PCWP and LAP may not be accurate measurements of volume when CPAP is utilized.

## 1. Background

The effects of positive pressure ventilation on hemodynamic parameters are known to be complex. They are determined by an integral relationship betweenvascular resistance and intrathoracic pressure [1, 2].

Positive pressure ventilation increases intrathoracic pressure (ITP), which increases right atrial (RA) pressure, leading to a decrease in venous return. Subsequently, this decreased right ventricle (RV) filling decreases left ventricle (LV) preload and allows for greater LV contraction with decreased energy expenditure. In patients who are volume overloaded, this could be beneficial. However, in hypovolemic patients, this may induce cardiovascular insufficiency [2, 3]. Therefore, knowledge of the volume status of mechanically ventilated patients is essential. On critically ill patients, pulmonary artery catheters (PACs) can be used for hemodynamic monitoring. Pulmonary capillary wedge pressure is reflective of left atrial pressure (LAP). Left atrial pressure is reflective of left ventricular end-diastolic pressure (LVEDP), which is a measure of preload, and preload is an estimation of volume. These relationships hold true when cardiac compliance is constant and pulmonary capillary pressure is greater than alveolar pressure [4].

Other effects of positive pressure ventilation can be less beneficial. For example, juxtacardiac ITP due to hyperexpanding lungs can decrease left ventricular diastolic compliance and subsequently impair LV contractility [2]. In some studies, this has been associated with decreased cardiac output [5]. Also, positive end expiratory pressure (PEEP) can induce regional hyperinflation, which compresses alveolar vessels and increases pulmonary vascular resistance (PVR), which can potentially lead to RV failure, or cor pulmonale [2]. 

Despite these hemodynamic effects, mechanical ventilation improves pulmonary gas exchange and restores arterial blood acid-base balance. There are many modes of mechanical respiratory support but airway pressure release ventilation (APRV) has offered clinical advantages for ventilator management of acute lung injury (ALI) and acute respiratory distress syndrome (ARDS) in comparison to conventional mechanical ventilation [6, 7]. APRV is a method of ventilation that uses continuous airway pressure in time-released cyclical fashion that was first described in 1987 [8]. This mode of ventilation is theoretically known for improved ventilation-perfusion matching through improved alveolar recruitment, leading to improved airway exchange. Many advantages of this mode have been described, such as: lower airway pressures, lower minute ventilation, minimal adverse effects on cardiocirculatory function, ability to spontaneously breathe throughout the entire ventilatory cycle, decrease of sedation use, and near elimination of neuromuscular blockade [6, 7, 9]. In addition, APRV is theoretically believed to improve cardiac output by lowering the right atrial pressure and improving preload due to decrease of pleural pressures and increase in abdominal pressure [9].

 The relationship between LAP and PCWP has not been formally assessed with APRV. In this study, we compare PCWP with LAP in a swine model while APRV mode of ventilation is used to establish the reliability of this measurement in estimating volume.

## 2. Method

Seven 30–45 kg male swine were sedated with propofol to facilitate tracheal intubation and instrumentation. However, muscle relaxants were not given to the animals so they were able to ventilate spontaneously. A pressure transducer was placed down the endotracheal tube and used to monitor mean airway pressure (Paw). Right and left femoral arterial and venous accesses were obtained to monitor mean arterial pressure (MAP) and central venous pressure (CVP), respectively. Then, a Medtronic 8 French transseptal sheath was introduced through atrial septum and connected to pressure transducer to continuously record LAP. Finally, pulmonary artery catheter was advanced until distal tip was placed into the pulmonary artery, radio-graphically below the level of the left atria, for measurement of PCWP at the end of expiration. All connections to the pressure transducer were zeroed and measurements confirmed. The pressure transducer was zeroed at the level of the atria prior to obtaining measurements. The animals were placed on conventional ventilation (assisted control) with tidal volume 6cc/kg and PEEP of 5 cm H_2_O. Mean PCWP and LAP were recorded as baseline values. APRV protocol initiated with changing CPAP-high from 10 to 35 cm H_2_O and CPAP-low varied from 5 to 30 cm H_2_O in 5 unit increments with a ratio of T_High_ to T_Low_ of 4 : 1. Hemodynamic parameters were recorded for 30 seconds after each APRV pressure change following a period of stabilization that ranged from 3 to 5 minutes. Linear regression analysis (Sigma Stat version 3.1) was employed to assess changes in mean PCWP and mean LAP with increases in high PEEP and at constant low PEEP (5 cm H_2_O). The relationship between mean PCWP (mmHg) and LAP (mmHg) was assessed by regression analysis for each animal to assess individual variability and all animals pooled with ARPV. *P* values <.05 were considered significant.

## 3. Results

**Figure 1:**
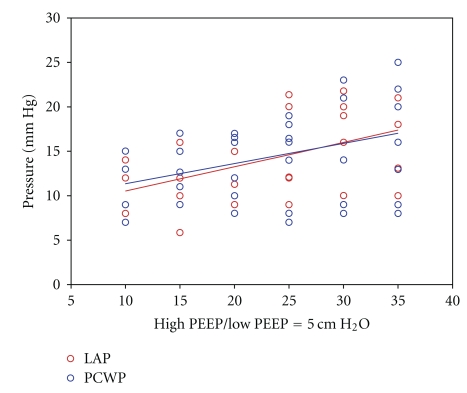
Illustration of variability in LAP and PCWP as high PEEP is increases with constant low PEEP of 5 cm H_2_O.

**Figure 2:**
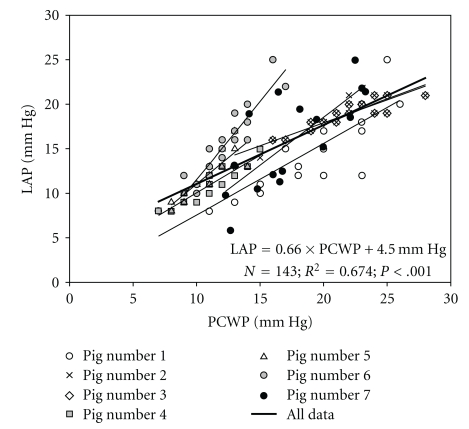
Regression data comparing left atrial pressure (LAP) to pulmonary capillary wedge pressure (PCWP).

With increasing levels of CPAP during APRV, mean PCWP and LAP increased in response to increasing airway pressure. Mean PCWP and LAP of the group were linearly related (LAP = 0.66*PCWP + 4.5 cm H_2_O, *R* ^2^ = 0.674, and *P* < .001) over a wide range of high and low CPAP during ARPV (Figure 1). The slope of this regression line differed with each individual animal (Figure 2). A Bland-Altman analysis also demonstrates agreement between mean PCWP and LAP over a 95% confidence interval (CI).

## 4. Discussion

To our knowledge, this is the first study to evaluate changes in cardiovascular parameters during increasing levels of CPAP with APRV mode of ventilation. The linear relationship between PCWP and LAP, conferred by two methods of agreement, demonstrates how PCWP is an accurate assessment of LAP with APRV mode of ventilation over CPAP ranges of 10 to 35 cm H_2_O. The individualized relationship between these values for each animal likely represents the unique cardiac and pulmonary compliance of each animal. 

**Figure 3:**
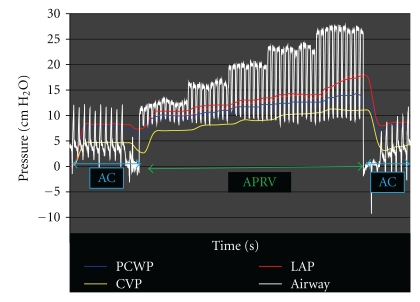
Representation of changes in PCWP and LAP in the same swine model as modes of ventilation change from AC to APRV and back to AC with return of both PCWP and LAP to baseline.

This experimental swine model also demonstrates that increasing levels of CPAP transpire to elevations of PCWP and LAP from baseline values. Since no interventions in volume were made during this assessment and these values returned to baseline when convention ventilation was restored, this suggests that the temporary elevation in these pressures was not caused by permanent volume change but by temporary pressure change (Figure 3). Also, when evaluating pressure changes during periods of T_High_ and T_Low_, there were no change in the readings of either PCWP or LAP and they remained elevated without drop to baseline.

There were several limitations to this study. First, the swine used had healthy, compliant lungs. APRV is normally used in patients with injured, less compliant lungs. One could argue that compliant lungs would be able to expand further with greater levels of PEEP and increase intrathoracic affects on cardiac compliance. Second, CPAP in APRV is commonly set to levels beyond the 35 cm H_2_O maximum used in this study. Therefore, it is unclear how greater CPAP would affect hemodynamic monitoring. Also, without monitoring ITP, we were unable to comment on how this variable affects hemodynamic monitoring. This limitation was due to the fact that attempts at placement of ITP in training models lead to pneumothoraces and attempts at placement were aborted in study model. Despite these limitations, the waveform tracings clearly demonstrate how increased positive pressure increases hemodynamic parameters without any changes in volume status. Future studies with animal models mimicking ARDS are planned to address these limitations and define the change in cardiac output and left ventricular chamber size with APRV.

## 5. Conclusion

PCWP accurately reflects LAP during APRV with increasing CPAP. However, PCWP and LAP may not be accurate estimates of volume. The poor correlation of PCWP to left ventricular end-diastolic volume in patients with elevated PEEP is a well-investigated finding under conventional modes of ventilation and appears to hold true with APRV. Whether this disparity is secondary to the hypothetical influences of ITP or cardiopulmonary compliance has yet to be demonstrated. 

## Abbreviations

ALI: Acute lung injury
ARDS: Acute respiratory distress syndrome
APRV: Airway pressure release ventilation
CVP: Central venous pressure
CPAP: Continuous positive airway pressure
ITP: Increases in intrathoracic pressure
LAP: Left atrial pressure
LV: Left ventricle
LVEDP: Left ventricular end-diastolic pressure
MAP: Mean arterial pressure
PAC: Pulmonary artery catheters
PEEP: Positive end-expiratory pressure
PCWP: Pulmonary capillary wedge pressure
RA: Right atrial
RV: Right ventricle
RVR: Right ventricular resistance.

## References

[1] Pinsky MR (1997). The hemodynamic consequences of mechanical ventilation: an evolving story. *Intensive Care Medicine*.

[2] Pinsky MR (2005). Cardiovascular issues in respiratory care. *Chest*.

[3] Luecke T, Pelosi P (2005). Clinical review: positive end-expiratory pressure and cardiac output. *Critical Care*.

[4] Marino P (2007). *The ICU Book*.

[5] Viquerat CE, Righetti A, Suter PM (1983). Biventricular volumes and function in patients with adult respiratory distress syndrome ventilated with PEEP. *Chest*.

[6] Habashi NM (2005). Other approaches to open-lung ventilation: airway pressure release ventilation. *Critical Care Medicine*.

[7] Siau C, Stewart TE (2008). Current role of high frequency oscillatory ventilation and airway pressure release ventilation in acute lung injury and acute respiratory distress syndrome. *Clinics in Chest Medicine*.

[8] Stock MC, Downs JB, Frolicher DA (1987). Airway pressure release ventilation. *Critical Care Medicine*.

[9] Kaplan LJ, Bailey H, Formosa V (2001). Airway pressure release ventilation increases cardiac performance in patients with acute lung injury/adult respiratory distress syndrome. *Critical Care*.

